# (4a*R*,6a*S*,10a*R*,10b*S*)-7,7,10a-Trimethyl-1,4,4a,5,6,6a,7,8,9,10,10a,10b-dodecahydro-2*H*-naphtho­[2,1-*c*]pyran (Pyamber)

**DOI:** 10.1107/S1600536811038694

**Published:** 2011-10-08

**Authors:** Gary B. Evans, Graeme J. Gainsford

**Affiliations:** a Carbohydrate Chemistry Group, Industrial Research Limited, PO Box 31-310, Lower Hutt, New Zealand

## Abstract

The crystal structure of the title compound, C_16_H_28_O, features C—H⋯O hydrogen bonds making *C*(6) zigzag chains along one 2_1_ screw axis. Within the limits of the data collection affected by crystal quality, the Hooft parameter gave correct indications of the known molecular chirality based on the single O atom anomalous dispersion in contrast to the indeterminate Flack value. Synthetic steps starting from manool are reported.

## Related literature

For details of the synthesis, see: Evans & Grant (1997 ▶); Grant *et al.* (1988 ▶); Vlad *et al.* (1978 ▶, 1983 ▶). For the related structure methyl 8,9-ep­oxy-12-oxo-13-oxototarane-14β-carboxyl­ate, see: Cambie *et al.* (1988 ▶). For a description of the Cambridge Structural Database, see: Allen (2002 ▶). For hydrogen-bond motifs, see: Bernstein *et al.* (1995 ▶). For determination of absolute configuration, see: Hooft *et al.* (2008 ▶).
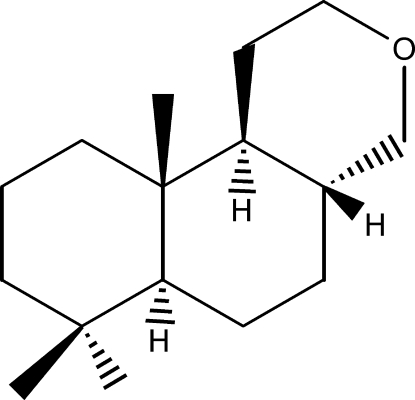


## Experimental

### 

#### Crystal data

C_16_H_28_O
*M* *_r_* = 236.38
Orthorhombic, 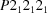

*a* = 7.3497 (2) Å
*b* = 11.1642 (3) Å
*c* = 17.0758 (12) Å
*V* = 1401.13 (11) Å^3^
*Z* = 4
Cu *K*α radiation
μ = 0.50 mm^−1^
*T* = 123 K
0.70 × 0.40 × 0.13 mm

#### Data collection

Rigaku Spider diffractometer
Absorption correction: multi-scan (*ABSCOR*; Higashi, 1995 ▶) *T*_min_ = 0.687, *T*_max_ = 1.0
5141 measured reflections
1969 independent reflections
1823 reflections with *I* > 2σ(*I*)
*R*_int_ = 0.033
θ_max_ = 58.9°

#### Refinement

*R*[*F* ^2^ > 2σ(*F* ^2^)] = 0.034
*wR*(*F* ^2^) = 0.091
*S* = 1.06
1969 reflections
158 parameters
H-atom parameters constrained
Δρ_max_ = 0.14 e Å^−3^
Δρ_min_ = −0.12 e Å^−3^
Absolute structure: Flack (1983 ▶), 801 Friedel pairs
Flack parameter: −0.4 (4)

### 

Data collection: *CrystalClear* (Rigaku, 2005 ▶); cell refinement: FSProcess in *PROCESS-AUTO* (Rigaku, 1998 ▶); data reduction: FSProcess in *PROCESS-AUTO*; program(s) used to solve structure: *SHELXS97* (Sheldrick, 2008 ▶); program(s) used to refine structure: *SHELXL97* (Sheldrick, 2008 ▶); molecular graphics: *ORTEP* in *WinGX* (Farrugia, 1999 ▶) and *Mercury* (Macrae *et al.*, 2008 ▶); software used to prepare material for publication: *SHELXL97* and *PLATON* (Spek, 2009 ▶).

## Supplementary Material

Crystal structure: contains datablock(s) global, I. DOI: 10.1107/S1600536811038694/zj2024sup1.cif

Structure factors: contains datablock(s) I. DOI: 10.1107/S1600536811038694/zj2024Isup2.hkl

Supplementary material file. DOI: 10.1107/S1600536811038694/zj2024Isup3.cml

Additional supplementary materials:  crystallographic information; 3D view; checkCIF report

## Figures and Tables

**Table 1:** Hydrogen-bond geometry (Å, °)

*D*—H⋯*A*	*D*—H	H⋯*A*	*D*⋯*A*	*D*—H⋯*A*
C6—H6*A*⋯O1^i^	0.99	2.54	3.474 (2)	158

Symmetry code: (i) 
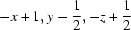
.

**Fig. 1.**
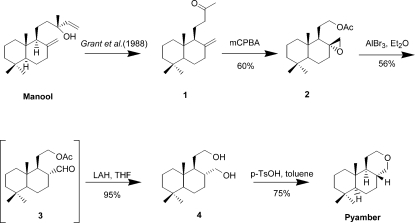
Chemical synthesis of Pyamber

**Fig. 2.**
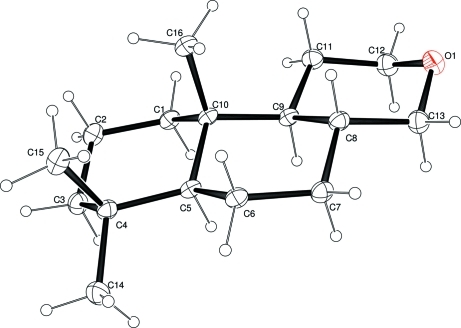
An ORTEP (Farrugia, 1999) view showing the asymmetric unit with 20% probabilility ellipsoids.

**Fig. 3.**
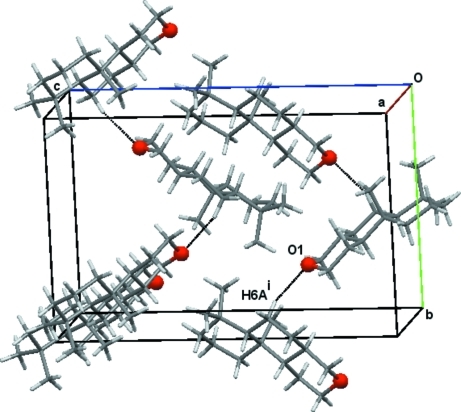
Mercury cell packing view (Macrae et al., 2008) showing the linking hydrogen bonds (dotted lines, Table 1). Symmetry (i) 1 - x, 1/2 + y, 1/2 - z.

## supplementary crystallographic information

### Comment

The title compound ("pyamber") was synthesized from methyl ketone 1, which is readily synthesized from manool in multi-gram quantites (Grant *et al.*, 1988), in 4 synthetic steps (Fig. 1). A Baeyer-Villager insertion reaction of neat 1 was achieved using mCPBA to afford the previously described acetate 2 in good yield (Evans & Grant, 1997). Treatment of 2 with aluminium bromide in anhydrous diethyl ether gave one major product, aldehyde 3, which was not characterized but then immediately reduced with lithium aluminium hydride to afford diol 4 in moderate yield for the two steps (Vlad *et al.*, 1978). Cyclization through dehydration was readily achieved under Dean and Stark conditions to afford crystalline pyamber in good yield, following chromatography; the analytical data was identical to that previously reported by Vlad *et al.* (1983).

The title compound, C_16_H_28_O, crystallizes with one independent molecule in the asymmetric unit (Fig. 2). Only confirmation of structure was required for this study, with the absolute configurations of C5(S), C8(*R*), C9(S) & C10(S) expected from the synthesis. The Flack parameter is hardly convincing, though the Hooft equivalent parameter (Hooft *et al.*, 2008) at -0.13 (17), with clear outliers removed [*PLATON* (Spek, 2009)], provides a probability of being false (P3) of 0.6 *x* 10^-9^ [P3(true) & P3(rac-twin) were 0.998,0.002 respectively]. The crystal quality/data collection also was not optimum (see experimental), but even including all measured data (2547 reflections) the Hooft equivalent parameter is -0.15 (17) while the Flack parameter changes to 0.2 (8).

There are very few structures reported with these same three fused six-membered rings (Allen, 2002. CSD version 5.32, with May 2011 update); each ring is in a standard chair conformation. The closest related compound is the epoxide structure methyl 8,9-epoxy-12-oxo-13-oxototarane-14β-carboxylate (Cambie *et al.*, 1988) reported in the inverted configuration. The molecules pack in zigzag chains along the b 2_1_ screw axis bound by one C—H···O hydrogen bond (Fig. 3, Table 1) described by the motif C(6) (Bernstein *et al.*, 1995). These chains are efficiently interlocked in the other two cell directiions *via* van der Waals interactions.

### Experimental

2-[(2*S*,4aS,8aS)-5,5,8a-Trimethyl-octahydro-1*H*-spiro[naphthalene -2,2'-oxirane]-1-yl]ethyl acetate (2): mCPBA (26.4 g, 50% b.w., 76.4 mmol) was added portionwise to a stirred solution of methyl ketone 1 (5 g, 19.1 mmol) in chloroform (150 ml). The round bottom flask was transferred to a rotavapor and the chloroform removed *in vacuo* at 40° C and the resulting paste left to rotate at 40° C overnight. The paste was redissolved in chloroform (200 ml) and stirred with calcium hydroxide (20 g) for 2 h, filtered through Celite&reg; and then concentrated *in vacuo*. The resulting oil was purified by flash chromatography on silica gel (1:4 ethyl acetate:petrol) to afford compound 2 (3.7 g, 66%). ^1^H and ^13^C NMR were identical to that described in the literature (Evans & Grant, 1997).

2-[(2*R*,4aS,8aR)-2-(hydroxymethyl)-5,5,8a-trimethyl-decahydronaphthalen-1-yl] ethan-1-ol (4): Aluminium bromide (3.7 g, 13.8 mmol) was added portionwise to a solution of epoxy acetate 2 (3.7 g, 12.6 mmol) in anhydrous diethyl ether under an inert atmosphere. The reaction was stirred for 15 min and then quenched with aqueous sodium hydroxide (15% b.w., 50 ml). The diethyl ether solution was diluted further with diethyl ether (150 ml) and the organic layer was washed with water (50 ml), brine (50 ml), dried (MgSO_4_), and concentrated *in vacuo* to afford an oily solid which was used in the next step without purification.

The oily residue was redissolved in THF (50 ml) and stirred overnight with lithium aluminium hydride (1 g, excess) under an inert atmosphere. The next morning the reaction was deemed to be complete by TLC and quenched with water (1 ml), aqueous sodium hydroxide (15% b.w., 1 ml), and water (3 ml), filtered through Celite&reg; and concentrated *in vacuo* to afford a solid residue. Purification of the crude residue was achieved by chromatography on silica gel (2:3 ethyl acetate:petrol) to afford diol 4 (1.80 g, 56%) as a crystalline solid. ^1^H NMR was identical to that previously described in the literature (Vlad *et al.*, 1978). ^13^C NMR (125 MHz, CDCl_3_) δ 65.0, 64.5, 54.9, 48.1, 42.3, 40.6, 39.1, 38.4, 33.4, 33.3, 30.5, 29.6, 21.9, 21.6, 18.8, 14.0.

(4aR,6aS,10aR,10bS)-7,7,10*a*-Trimethyl-octahydro-1*H*-naphtho[2,1-*c*]pyran (Pyamber): *p*-TsOH.H_2_O (1.6 g, 8.4 mmol) was added portionwise to a stirred suspension of 4 (1.8 g, 7.1 mmol) in anhydrous toluene under an inert atmosphere. The resulting mixture was stirred at reflux under Dean and Stark conditions for 2 h, cooled to ambient temperature and then stirred with solid NaHCO_3_. After 1 h the suspension was filtered and the filtrate concentrated *in vacuo*. The crude residue was purified by flash chromatography on silica gel (1:24 ethyl acetate:petrol) to afford pyamber (1.48 g, 88%) as a white crystalline solid, m.p. 52–53° C. ^1^H NMR was identical to that described in the literature (Vlad *et al.*, 1983). ^13^C NMR (125 MHz, CDCl_3_) δ 74.2, 69.1, 55.5, 54.1, 42.4, 38.5, 36.3, 35.9, 33.5, 33.3, 29.4, 25.2, 21.9, 21.0, 18.8, 14.3. C_16_H_28_O requires C, 81.29; H, 11.94. Found C, 81.10; H, 11.69.

### Refinement

After structural refinement it was noted that high angle data (d < 0.90 Å) had systematically F_o_**2 << F_c_**2; this reflected the poor profiles noticed for higher angle data, but may have also been affected by minor movement (some icing was noted). As a consequence data was limited to that within the d resolution of 0.90 Å. A further 8 reflections (one at low angle) within this shell were clearly outliers and so were excluded.

The methyl H atoms were constrained to an ideal geometry (C—H = 0.98 Å) with *U*_iso_(H) = 1.5*U*_eq_(C), but were allowed to rotate freely about the adjacent C—C bonds. All other H atoms were placed in geometrically idealized positions and constrained to ride on their parent atoms with C—H distances of 0.99Å and *U*_iso_(H) = 1.2*U*_eq_(C).

### Crystal data

**Table:**

C_16_H_28_O	*F*(000) = 528
*M**_r_* = 236.38	*D*_x_ = 1.121 Mg m^−^^3^
Orthorhombic, *P*2_1_2_1_2_1_	Cu *K*α radiation, λ = 1.54178 Å
Hall symbol: P 2ac 2ab	Cell parameters from 484 reflections
*a* = 7.3497 (2) Å	θ = 6.6–72.0°
*b* = 11.1642 (3) Å	µ = 0.50 mm^−^^1^
*c* = 17.0758 (12) Å	*T* = 123 K
*V* = 1401.13 (11) Å^3^	Needle, colourless
*Z* = 4	0.70 × 0.40 × 0.13 mm

### Data collection

**Table:**

Rigaku Spider diffractometer	1969 independent reflections
Radiation source: Rigaku MM007 rotating anode	1823 reflections with *I* > 2σ(*I*)
Rigaku VariMax-HF Confocal Optical System	*R*_int_ = 0.033
Detector resolution: 10 pixels mm^-1^	θ_max_ = 58.9°, θ_min_ = 6.5°
ω–scans	*h* = −8→5
Absorption correction: multi-scan (*ABSCOR*; Higashi, 1995)	*k* = −10→12
*T*_min_ = 0.687, *T*_max_ = 1.0	*l* = −12→18
5141 measured reflections	

### Refinement

**Table:**

Refinement on *F*^2^	Hydrogen site location: inferred from neighbouring sites
Least-squares matrix: full	H-atom parameters constrained
*R*[*F*^2^ > 2σ(*F*^2^)] = 0.034	*w* = 1/[σ^2^(*F*_o_^2^) + (0.0509*P*)^2^ + 0.0905*P*] where *P* = (*F*_o_^2^ + 2*F*_c_^2^)/3
*wR*(*F*^2^) = 0.091	(Δ/σ)_max_ < 0.001
*S* = 1.06	Δρ_max_ = 0.14 e Å^−^^3^
1969 reflections	Δρ_min_ = −0.12 e Å^−^^3^
158 parameters	Extinction correction: *SHELXL*, Fc^*^=kFc[1+0.001xFc^2^λ^3^/sin(2θ)]^-1/4^
0 restraints	Extinction coefficient: 0.0066 (8)
Primary atom site location: structure-invariant direct methods	Absolute structure: Flack (1983), 780 Friedel pairs
Secondary atom site location: difference Fourier map	Flack parameter: −0.4 (4)

### Special details

**Table:**

Geometry. All e.s.d.'s (except the e.s.d. in the dihedral angle between two l.s. planes) are estimated using the full covariance matrix. The cell e.s.d.'s are taken into account individually in the estimation of e.s.d.'s in distances, angles and torsion angles; correlations between e.s.d.'s in cell parameters are only used when they are defined by crystal symmetry. An approximate (isotropic) treatment of cell e.s.d.'s is used for estimating e.s.d.'s involving l.s. planes.
Refinement. Refinement of *F*^2^ against ALL reflections. The weighted *R*-factor *wR* and goodness of fit *S* are based on *F*^2^, conventional *R*-factors *R* are based on *F*, with *F* set to zero for negative *F*^2^. The threshold expression of *F*^2^ > σ(*F*^2^) is used only for calculating *R*-factors(gt) *etc*. and is not relevant to the choice of reflections for refinement. *R*-factors based on *F*^2^ are statistically about twice as large as those based on *F*, and *R*- factors based on ALL data will be even larger.

### Fractional atomic coordinates and isotropic or equivalent isotropic displacement parameters (Å^2^)

**Table:**

	*x*	*y*	*z*	*U*_iso_*/*U*_eq_	
O1	0.40780 (17)	0.74765 (10)	0.29584 (7)	0.0466 (4)	
C1	0.1359 (2)	0.60896 (15)	0.02512 (10)	0.0351 (4)	
H1A	0.0226	0.6177	0.0560	0.042*	
H1B	0.1734	0.6899	0.0078	0.042*	
C2	0.0957 (2)	0.53268 (15)	−0.04708 (10)	0.0393 (4)	
H2A	0.0458	0.4544	−0.0305	0.047*	
H2B	0.0030	0.5732	−0.0797	0.047*	
C3	0.2675 (2)	0.51266 (16)	−0.09533 (10)	0.0390 (4)	
H3A	0.3091	0.5906	−0.1164	0.047*	
H3B	0.2377	0.4604	−0.1404	0.047*	
C4	0.4228 (2)	0.45576 (14)	−0.04894 (10)	0.0348 (4)	
C5	0.4558 (2)	0.53078 (14)	0.02654 (9)	0.0313 (4)	
H5	0.4936	0.6113	0.0067	0.038*	
C6	0.6176 (2)	0.48888 (15)	0.07615 (9)	0.0347 (4)	
H6A	0.5872	0.4114	0.1012	0.042*	
H6B	0.7242	0.4760	0.0417	0.042*	
C7	0.6663 (2)	0.57965 (16)	0.13907 (10)	0.0368 (4)	
H7A	0.7145	0.6529	0.1138	0.044*	
H7B	0.7635	0.5461	0.1727	0.044*	
C8	0.5036 (2)	0.61297 (15)	0.19000 (10)	0.0352 (4)	
H8	0.4655	0.5405	0.2202	0.042*	
C9	0.3432 (2)	0.65343 (15)	0.13913 (9)	0.0322 (4)	
H9	0.3881	0.7234	0.1081	0.039*	
C10	0.2851 (2)	0.55687 (14)	0.07831 (9)	0.0305 (4)	
C11	0.1914 (2)	0.70176 (16)	0.19186 (10)	0.0408 (5)	
H11A	0.0955	0.7387	0.1591	0.049*	
H11B	0.1360	0.6347	0.2214	0.049*	
C12	0.2640 (3)	0.79350 (16)	0.24854 (11)	0.0462 (5)	
H12A	0.3090	0.8637	0.2189	0.055*	
H12B	0.1638	0.8209	0.2829	0.055*	
C13	0.5560 (2)	0.71069 (16)	0.24787 (10)	0.0422 (5)	
H13A	0.6555	0.6809	0.2818	0.051*	
H13B	0.6026	0.7808	0.2186	0.051*	
C14	0.5941 (3)	0.46036 (17)	−0.10071 (10)	0.0472 (5)	
H14A	0.6897	0.4108	−0.0773	0.071*	
H14B	0.6367	0.5433	−0.1047	0.071*	
H14C	0.5649	0.4300	−0.1531	0.071*	
C15	0.3821 (3)	0.32245 (14)	−0.03348 (11)	0.0431 (5)	
H15A	0.3920	0.2775	−0.0826	0.065*	
H15B	0.2586	0.3142	−0.0125	0.065*	
H15C	0.4698	0.2909	0.0045	0.065*	
C16	0.2115 (2)	0.44509 (14)	0.12111 (10)	0.0372 (4)	
H16A	0.1403	0.3964	0.0845	0.056*	
H16B	0.1338	0.4702	0.1648	0.056*	
H16C	0.3135	0.3978	0.1412	0.056*	

### Atomic displacement parameters (Å^2^)

**Table:**

	*U*^11^	*U*^22^	*U*^33^	*U*^12^	*U*^13^	*U*^23^
O1	0.0496 (8)	0.0512 (7)	0.0389 (6)	0.0050 (7)	−0.0014 (6)	−0.0043 (6)
C1	0.0215 (10)	0.0358 (9)	0.0480 (10)	0.0030 (8)	0.0000 (8)	0.0003 (8)
C2	0.0313 (10)	0.0395 (9)	0.0473 (10)	0.0030 (9)	−0.0102 (8)	0.0012 (8)
C3	0.0339 (10)	0.0411 (9)	0.0421 (9)	0.0012 (8)	−0.0040 (8)	−0.0042 (8)
C4	0.0247 (9)	0.0394 (9)	0.0404 (9)	−0.0013 (8)	0.0005 (8)	−0.0033 (8)
C5	0.0241 (9)	0.0292 (9)	0.0406 (9)	−0.0025 (7)	0.0020 (8)	0.0030 (8)
C6	0.0232 (9)	0.0392 (9)	0.0417 (9)	0.0015 (8)	0.0016 (8)	0.0005 (8)
C7	0.0257 (10)	0.0428 (10)	0.0418 (9)	0.0005 (8)	−0.0036 (8)	0.0030 (8)
C8	0.0286 (9)	0.0396 (10)	0.0373 (9)	−0.0022 (8)	−0.0012 (7)	0.0039 (8)
C9	0.0254 (9)	0.0318 (9)	0.0392 (9)	−0.0021 (7)	0.0031 (7)	0.0009 (8)
C10	0.0214 (9)	0.0314 (9)	0.0389 (9)	−0.0025 (7)	0.0001 (8)	0.0024 (7)
C11	0.0376 (11)	0.0419 (10)	0.0429 (9)	0.0021 (9)	0.0034 (9)	−0.0005 (9)
C12	0.0456 (11)	0.0461 (11)	0.0468 (10)	0.0047 (10)	0.0031 (10)	−0.0040 (9)
C13	0.0409 (11)	0.0449 (11)	0.0407 (9)	0.0009 (9)	−0.0022 (10)	−0.0027 (9)
C14	0.0387 (11)	0.0596 (12)	0.0433 (10)	0.0021 (10)	0.0036 (9)	−0.0082 (9)
C15	0.0340 (11)	0.0349 (9)	0.0604 (11)	0.0052 (8)	−0.0052 (10)	−0.0077 (9)
C16	0.0292 (10)	0.0339 (9)	0.0484 (10)	−0.0016 (8)	0.0026 (8)	0.0015 (8)

### Geometric parameters (Å, °)

**Table:**

O1—C13	1.424 (2)	C8—C13	1.522 (2)
O1—C12	1.425 (2)	C8—C9	1.532 (2)
C1—C2	1.527 (2)	C8—H8	1.0000
C1—C10	1.538 (2)	C9—C11	1.532 (2)
C1—H1A	0.9900	C9—C10	1.557 (2)
C1—H1B	0.9900	C9—H9	1.0000
C2—C3	1.524 (2)	C10—C16	1.544 (2)
C2—H2A	0.9900	C11—C12	1.507 (2)
C2—H2B	0.9900	C11—H11A	0.9900
C3—C4	1.528 (2)	C11—H11B	0.9900
C3—H3A	0.9900	C12—H12A	0.9900
C3—H3B	0.9900	C12—H12B	0.9900
C4—C14	1.539 (2)	C13—H13A	0.9900
C4—C15	1.541 (2)	C13—H13B	0.9900
C4—C5	1.556 (2)	C14—H14A	0.9800
C5—C6	1.533 (2)	C14—H14B	0.9800
C5—C10	1.562 (2)	C14—H14C	0.9800
C5—H5	1.0000	C15—H15A	0.9800
C6—C7	1.520 (2)	C15—H15B	0.9800
C6—H6A	0.9900	C15—H15C	0.9800
C6—H6B	0.9900	C16—H16A	0.9800
C7—C8	1.525 (2)	C16—H16B	0.9800
C7—H7A	0.9900	C16—H16C	0.9800
C7—H7B	0.9900		
			
C13—O1—C12	110.20 (12)	C11—C9—C8	109.31 (13)
C2—C1—C10	113.79 (13)	C11—C9—C10	115.89 (13)
C2—C1—H1A	108.8	C8—C9—C10	112.64 (13)
C10—C1—H1A	108.8	C11—C9—H9	106.1
C2—C1—H1B	108.8	C8—C9—H9	106.1
C10—C1—H1B	108.8	C10—C9—H9	106.1
H1A—C1—H1B	107.7	C1—C10—C16	109.57 (13)
C3—C2—C1	110.99 (13)	C1—C10—C9	109.12 (12)
C3—C2—H2A	109.4	C16—C10—C9	109.88 (12)
C1—C2—H2A	109.4	C1—C10—C5	108.01 (11)
C3—C2—H2B	109.4	C16—C10—C5	113.50 (13)
C1—C2—H2B	109.4	C9—C10—C5	106.64 (12)
H2A—C2—H2B	108.0	C12—C11—C9	111.05 (14)
C2—C3—C4	113.55 (13)	C12—C11—H11A	109.4
C2—C3—H3A	108.9	C9—C11—H11A	109.4
C4—C3—H3A	108.9	C12—C11—H11B	109.4
C2—C3—H3B	108.9	C9—C11—H11B	109.4
C4—C3—H3B	108.9	H11A—C11—H11B	108.0
H3A—C3—H3B	107.7	O1—C12—C11	112.49 (15)
C3—C4—C14	107.42 (13)	O1—C12—H12A	109.1
C3—C4—C15	110.21 (14)	C11—C12—H12A	109.1
C14—C4—C15	106.83 (14)	O1—C12—H12B	109.1
C3—C4—C5	108.80 (13)	C11—C12—H12B	109.1
C14—C4—C5	109.28 (13)	H12A—C12—H12B	107.8
C15—C4—C5	114.09 (14)	O1—C13—C8	112.80 (13)
C6—C5—C4	114.48 (12)	O1—C13—H13A	109.0
C6—C5—C10	111.54 (11)	C8—C13—H13A	109.0
C4—C5—C10	116.34 (12)	O1—C13—H13B	109.0
C6—C5—H5	104.3	C8—C13—H13B	109.0
C4—C5—H5	104.3	H13A—C13—H13B	107.8
C10—C5—H5	104.3	C4—C14—H14A	109.5
C7—C6—C5	111.71 (13)	C4—C14—H14B	109.5
C7—C6—H6A	109.3	H14A—C14—H14B	109.5
C5—C6—H6A	109.3	C4—C14—H14C	109.5
C7—C6—H6B	109.3	H14A—C14—H14C	109.5
C5—C6—H6B	109.3	H14B—C14—H14C	109.5
H6A—C6—H6B	107.9	C4—C15—H15A	109.5
C6—C7—C8	112.41 (13)	C4—C15—H15B	109.5
C6—C7—H7A	109.1	H15A—C15—H15B	109.5
C8—C7—H7A	109.1	C4—C15—H15C	109.5
C6—C7—H7B	109.1	H15A—C15—H15C	109.5
C8—C7—H7B	109.1	H15B—C15—H15C	109.5
H7A—C7—H7B	107.9	C10—C16—H16A	109.5
C13—C8—C7	110.28 (13)	C10—C16—H16B	109.5
C13—C8—C9	110.59 (13)	H16A—C16—H16B	109.5
C7—C8—C9	110.61 (13)	C10—C16—H16C	109.5
C13—C8—H8	108.4	H16A—C16—H16C	109.5
C7—C8—H8	108.4	H16B—C16—H16C	109.5
C9—C8—H8	108.4		
			
C10—C1—C2—C3	−56.49 (18)	C2—C1—C10—C9	168.00 (13)
C1—C2—C3—C4	56.36 (18)	C2—C1—C10—C5	52.44 (17)
C2—C3—C4—C14	−170.86 (13)	C11—C9—C10—C1	58.17 (17)
C2—C3—C4—C15	73.11 (18)	C8—C9—C10—C1	−174.92 (12)
C2—C3—C4—C5	−52.67 (18)	C11—C9—C10—C16	−61.98 (18)
C3—C4—C5—C6	−175.92 (12)	C8—C9—C10—C16	64.93 (16)
C14—C4—C5—C6	−58.92 (17)	C11—C9—C10—C5	174.60 (13)
C15—C4—C5—C6	60.59 (18)	C8—C9—C10—C5	−58.48 (15)
C3—C4—C5—C10	51.59 (17)	C6—C5—C10—C1	174.96 (12)
C14—C4—C5—C10	168.59 (13)	C4—C5—C10—C1	−51.21 (17)
C15—C4—C5—C10	−71.91 (18)	C6—C5—C10—C16	−63.35 (17)
C4—C5—C6—C7	168.28 (13)	C4—C5—C10—C16	70.48 (16)
C10—C5—C6—C7	−56.98 (17)	C6—C5—C10—C9	57.78 (16)
C5—C6—C7—C8	53.70 (17)	C4—C5—C10—C9	−168.39 (13)
C6—C7—C8—C13	−175.75 (14)	C8—C9—C11—C12	50.94 (18)
C6—C7—C8—C9	−53.10 (19)	C10—C9—C11—C12	179.51 (14)
C13—C8—C9—C11	−50.14 (17)	C13—O1—C12—C11	60.46 (18)
C7—C8—C9—C11	−172.61 (13)	C9—C11—C12—O1	−56.93 (18)
C13—C8—C9—C10	179.51 (13)	C12—O1—C13—C8	−60.03 (18)
C7—C8—C9—C10	57.04 (17)	C7—C8—C13—O1	178.54 (14)
C2—C1—C10—C16	−71.65 (16)	C9—C8—C13—O1	55.88 (17)

### Hydrogen-bond geometry (Å, °)

**Table:**

*D*—H···*A*	*D*—H	H···*A*	*D*···*A*	*D*—H···*A*
C6—H6A···O1^i^	0.99	2.54	3.474 (2)	158

Symmetry codes: (i) −*x*+1, *y*−1/2, −*z*+1/2.
